# Biological characteristics of CD133^+^ cells in nasopharyngeal carcinoma

**DOI:** 10.3892/or.2013.2408

**Published:** 2013-04-22

**Authors:** HUI-WEN ZHUANG, TING-TING MO, WEI-JIAN HOU, GUAN-XIA XIONG, XIAO-LIN ZHU, QING-LING FU, WEI-PING WEN

**Affiliations:** Otorhinolaryngology Hospital, The First Affiliated Hospital, Sun Yat-sen University, Guangzhou, Guangdong 510080, P.R. China

**Keywords:** nasopharyngeal carcinoma, cancer stem cells, CD133

## Abstract

Cancer stem cells are regarded as the cause of tumour formation and recurrence in nasopharyngeal carcinoma (NPC). However, ideal surface markers for stem cells in NPC remain unidentified. In the present study, we investigated the expression of CD133, Nanog and Sox2 in the nasopharyngeal carcinoma cell line CNE2 and primarily cultured NPC cells using immunofluorescence or flow cytometry. A cell population with a CD133^+^ phenotype was enriched using magnetic-activated cell sorting technology. We demonstrated that CD133^+^ cells exhibited a strong potential for self-renewal, proliferation and differentiation and a greater potential for *in vivo* tumour formation in nude mice compared to CD133^−^ cells, although the percentage of CD133^+^ cells was small. However, the specific marker antigens Nanog and Sox2 were simultaneously expressed in normal cancer stem cells. Our results showed that CD133 can serve as a specific surface marker for nasopharyngeal cancer stem cells.

## Introduction

Recently, a growing number of studies have shown that cancer is a stem cell disease (1). Cancer stem cells possess unlimited potential for self-renewal and differentiation and exhibit high tumourigenicity and drug resistance. Compared with other cell types in tumours, cancer stem cells exhibit a greater capacity for proliferation, differentiation and invasion. They have also been shown to tolerate radiation and chemotherapy. These cells are not only the origin of the tumour cell population but are also the cause of tumour metastasis and recurrence (1). Under the guidance of the stem cell theory, researchers have used various methods to obtain and identify cancer stem cells in leukaemia, breast cancer, brain glioblastoma, prostate cancer, pancreatic cancer, colon cancer, ovarian cancer, liver cancer, lung cancer and other tumours (2–11). However, no systematic testing has been developed to explore the ideal surface markers for stem cells in nasopharyngeal carcinoma (NPC), which is one of the most common malignancies in Southeast Asia. Because of the scarcity of available human NPC tissues and the difficulties associated with collecting them, studies investigating stem cells within primary tumour cell populations have not previously been reported.

The CD133 antigen is a glycoprotein with a molecular weight of 117 kDa exhibiting 5 transmembrane domains (12). CD133 was originally reported as a specific marker for stem cells (13); however, subsequent studies have found it to be expressed in various tumour cell lines. Tumour cells expressing CD133 have been confirmed to be stem cells in human brain tumours, colon cancer, prostate cancer, liver cancer, melanoma, pancreatic cancer and other types of solid tumours (8,14–19), suggesting that CD133 may be a broad-spectrum marker of cancer stem cells (20).

In the present study, a cell population with a CD133^+^ phenotype from the NPC cell line and xenograft tumours was isolated using magnetic activated cell sorting (MACS) technology. The proliferation, differentiation of CD133^+^ cells and the tumourigenicity of CD133^+^ cells in nude mice were further investigated.

## Materials and methods

The present animal study was approved by the Ethics Committee of the First Hospital Affiliated of Sun Yat-sen University (no. 2009–01, 2009–02). The study conformed to the provisions of the Declaration of Helsinki.

### Cell culture

The CNE2 cell line was obtained from the Cancer Centre, Sun Yat-sen University (21). The cell culture medium used was RPMI-1640 (Guangzhou Land Bio, China) supplemented with 5% fetal calf serum (HyClone, USA). The cells were cultured in a water-saturated atmosphere under 5% CO_2_ at 37°C. Conversely, we separated tumour tissues from 20 NPC patients and performed a primary culture *in vitro*. Keratinocyte-SFM medium (Gibco, USA) was used to culture these primary human NPC cells. Features of the growth pattern of primary human NPC cells were observed. Flow cytometry was performed to quantify the expression of CD133 (Miltenyi, Germany) on the surface of NPC cells. The test was repeated 3 times.

### CD133 cell sorting using immunomagnetic beads

A single cell suspension of ~1×10^8^ CNE2 cells was used for cell sorting. Cells were incubated with CD133/l immunomagnetic beads (Miltenyi) for 30 min at 4°C. For magnetic separation, a MACS cell separation column was used to retain the positive cells linked with the beads. The CD133^+^ cells obtained from the column were centrifuged and resuspended in RPMl-1640 (with 5% inactivated foetal calf serum) or serum-free medium. The purity of CD133^+^ and CD133^−^ cells was evaluated by standard flow cytometric analysis. CD133^+^ and CD133^−^ cells were harvested, and their characteristics were determined for activities of proliferation, sphere formation and differentiation.

### Immunofluorescence

Slides with CD133^+^ and CD133^−^ cells were immersed in PBS for 5 min, and then were permeabilised with 0.1% Triton X-100 for 10 min. After washing with PBS 3 times for 5 min, the CD133^+^ and CD133^−^ cells were blocked with 5% BSA (HyClone, USA) at 37°C for 30 min. The cells were then incubated with primary antibodies, including Oct3/4 (Santa Cruz Biotechnology, Inc. Santa Cruz, CA, USA), Nanog (Abgent, USA) and Sox2 (Abcam, UK), in 5% BSA at 4°C for 16 h. The cells were then incubated with fluorescent secondary antibodies diluted with 1% BSA at 37°C for 60 min. Finally, the cell nuclei were stained with 4′,6-diamidino-2-phenylindole (DAPI) (Qiyun Biotechnology, Co., Ltd., China) (1:200), and the slides were visualised using fluorescence microscopy (IX71; Olympus, Japan).

### Proliferation assay

The proliferation of the cells was detected using an 3-(4,5-dimethylthiazol-2-yl)-2,5-diphenyltetrazolium bromide (MTT) assay on days 1, 3, 5 and 7. Three groups (including CD133^+^, CD133^−^ cells and unsorted cells) were plated onto 96-well plates (2,000 cells in 0.2 ml of cell culture medium/well). A blank well containing medium alone was used as the control. The cells were then incubated with MTT (5 mg/ml; Sigma, USA) in 20 μl at 4 h before the collection. The culture medium was finally removed, and 150 μl DMSO was added into the well. After shaking thoroughly for 10 min, the plates were read for the absorbance in an enzyme immunoassay instrument at 570 nm. Six wells were analysed for each group.

### Sphere formation

For subculturing of suspended cell spheres, CD133^+^ cells and CD133^−^ cells (1×10^3^/ml) were cultured in 6-well plates in serum-free DMEM-F12 medium with bFGF (10 ng/ml), EGF (20 ng/ml), B27 (10 μl/ml) and insulin (5 μg/ml). The cells were cultured under conditions of 5% CO_2_ at 37°C for 3 days, and the culture medium was replaced every other day.

### Differentiation assay

CD133^+^ cells obtained by immunomagnetic bead sorting were cultured in 6-well plates. On days 0, 3, 10, 15 and 21, the percentages of CD133^+^ cells were further determined by flow cytometry. The experiment was repeated 3 times, and the average values were calculated.

### Cell cycle phase distribution

A total of 1×10^6^ CD133^+^ and CD133^−^ cells were centrifuged at 1,000 rpm for 5 min, resuspended in 0.2 ml PBS and then fixed in l ml of 70% ethanol at 4°C for 16 h. After washing with PBS, the cells were then incubated with 300 μl of DNA dye at room temperature for 30 min. Cell cycle status was assessed by flow cytometry (Elite; Beckman-Coulter, USA). The relative proportions of cells in the G0/G1, S and G2/M phases were analysed, and the percentages of cells in each phase were calculated. The cell proliferation index (Pla) of each subset was calculated according to the following equation: Pla = 100 × (S + G2/M)/(G0/Gl + S + G2/M).

### Tumourigenicity in animals

Twelve male 4- to 6-week-old nude BALB/c mice from the Animal Centre at Sun Yat-sen University were used. Six mice received a subcutaneous inoculum of 1×10^4^ CD133^+^ cells in the upper right dorsum, and the other six mice received a transplantation of 1×10^5^ CD133^+^ cells. CD133^−^ cells with a similar cell density (either 1×10^4^ or 1×10^5^) were subcutaneously inoculated into the upper left dorsum of the mice. At 4 weeks after the transplantation, the xenograft tumours were collected and fixed in 4% paraformaldehyde for H&E staining or were incubated in explant tissue cultures with 3 ml of keratinocyte-SFM medium (Gibco) for cell culture. Finally, the expression of CD133 in the xenograft tumour cells was assessed by flow cytometry.

### CD133^+^ in primary human NPC cells

For the CD133^+^ cells isolated from primary human NPC cells, the expression, proliferation, differentiation and tumourigenicity of the CD133 cells were assessed as described above. Immunohistochemical analyses using a rat cytokeratin (CK) primary antibody against human cells and keratin monoclonal antibodies (Wuhan Boster Biological Technology, Ltd., Wuhan, China) were performed to identify the source of the cells.

### Statistical analysis

The statistical software SPSS11.0 was applied for data processing. Measured data are expressed as the mean ± SD. Independent sample t-test was used for performing comparisons between two groups. One-way ANOVA with Tamhane was used for comparisons among more than two groups. A P-value of 0.05 was considered to indicate a statistically significant result. The Chi-square test was used to compare the relative tumourigenicity of the 2 tumour cell subsets in the nude mice.

## Results

### CD133^+^ cells in CNE2 cells

**Figure 1:**
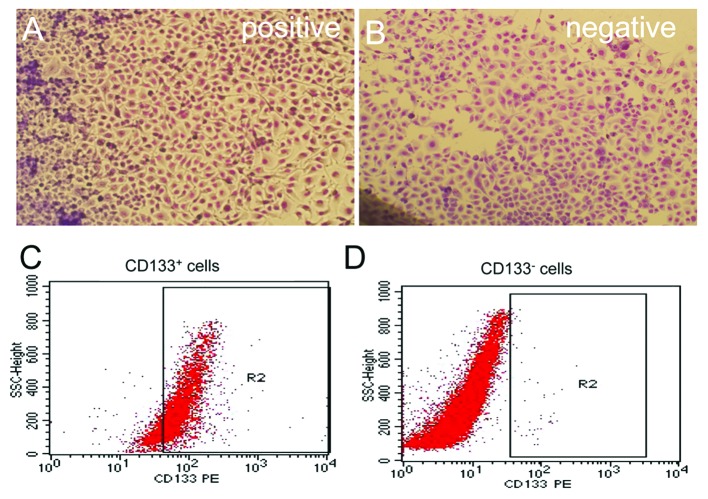
Characteristics of the CD133^+^ and CD133^−^ cells determined by histology and flow cytometry. (A and B) The morphology of cells under Giemsa staining (x100). (A) CD133^+^ cells were spindle-shaped and flat, with abundant cytoplasm and large nuclei, showing typical morphology for tumour cells. (B) CD133^−^ cells. (C and D) CD133^+^ and CD133^−^ cell subsets were assessed by flow cytometry. R2, positive cells.

Three independent measurements for the flow cytometric analysis detected CD133 expression rates of 3.23, 3.75 and 3.09%, with an average of 3.36±0.35%. After sorting, CD133^+^ and CD133^−^ tumour cells were cultured in RPMI-1640 medium containing 5% fetal calf serum. The CD133^+^ cells grew faster than the CD133^−^ cells. There were no significant differences for Giemsa staining between the 2 subsets of cells. After immunomagnetic sorting with CD133 beads, the cells were analysed by flow cytometry. Three measurements for CD133 expression in the CD133^+^-sorted population indicated a high CD133^+^ expression rate of 89.15±7.80%. However, the CD133^+^ expression rate in the CD133^−^-sorted population was only 0.23±0.04% (Fig. 1).

### Expression of cellular markers as detected by immunofluorescence

**Figure 2:**
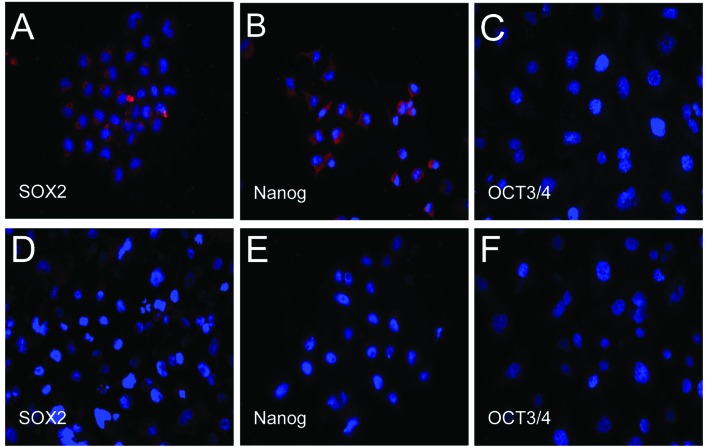
Expression of the stem/progenitor markers, Oct3/4, Nanog and Sox2, in CD133^+^ and CD133^−^ cells. (A-C) CD133^+^ cells. (D-F) CD133^−^ cells. Red, Oct3/4, Nanog and Sox2 staining; blue, 4′,6-diamidino-2-phenylindole (DAPI).

We found that both transcription factors, Nanog and Sox2, the main regulators of human embryonic stem cell pluripotency and self-renewal capacities, but not Oct3/4, were expressed in CD133^+^ cells. However, only weak expression of Nanog and Sox2, but no expression of Oct3/4, was observed in the CD133^−^ cells (Fig. 2).

### Proliferation assay

**Figure 3:**
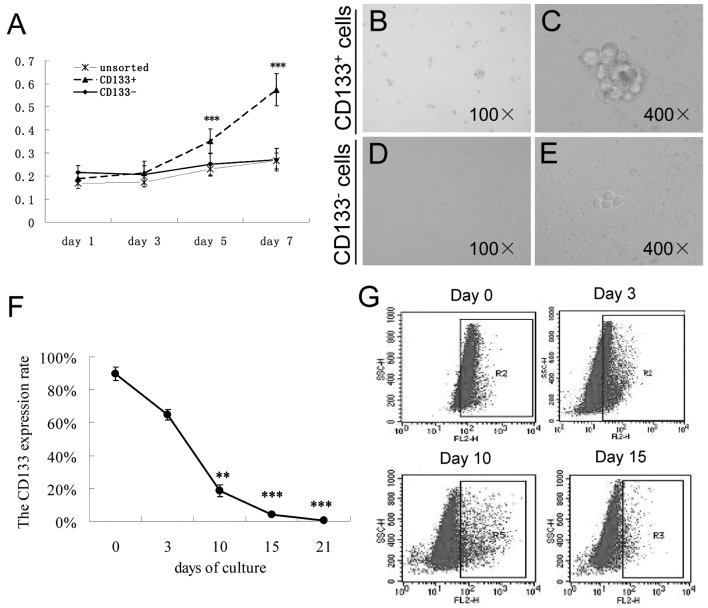
Characteristics of the CD133^+^ cells. (A) The growth curves of CD133^+^, CD133^−^ and unsorted CNE2 cells. ^***^P<0.001 compared to day 1. (B and C) Sphere formation activity of CD133^+^ cells in serum-free medium. (D and E) Sphere formation activity of CD133^−^ cells in serum-free medium. (F) The CD133 expression rate in sorted CD133^+^ CNE2 cells. ^**^P<0.01, ^***^P<0.001 compared to day 0. (G) Percentage of CD133^+^ cells detected by flow cytometry on different days of cultures.

CD133^+^ cells exhibited a high proliferation rate when compared with the rate in the CD133^−^ and unsorted cells on days 5 (0.351±0.012) and 7 (0.573±0.0165) (both P<0.001), but not on days 1 and 3 (Fig. 3A).

### Suspension cell culture

The CD133^+^ and CD133^−^ cells obtained after immunomagnetic cell sorting were harvested and cultured in serum-free culture medium containing various growth factors. After 3 days, many individual cells in the CD133^+^ cell culture were observed to survive and proliferate in suspension. These cells gradually formed sphere colonies with different sizes and irregular shapes. However, most of the cells finally died in the same serum-free medium in the CD133^−^ cell culture. Only a few CD133^−^ cells adhered to the wall and grew slowly; additionally, no clear sphere colony was found (Fig. 3B-E).

### Differentiation of CD133^+^ cells

Using flow cytometry, we found that CD133 expression decreased from 89.15±7.80% on day 0 to 18.4±3.7% on day 10 (P<0.01), 4±0.45% on day 15 (P<0.001) and 0.37±0.10% on day 21 (P<0.001) in the culture system (Fig. 3F and G). This finding indicates that CD133^+^ cells have the potential to differentiate into other types of cells.

### Cell cycle distribution

**Figure 4:**
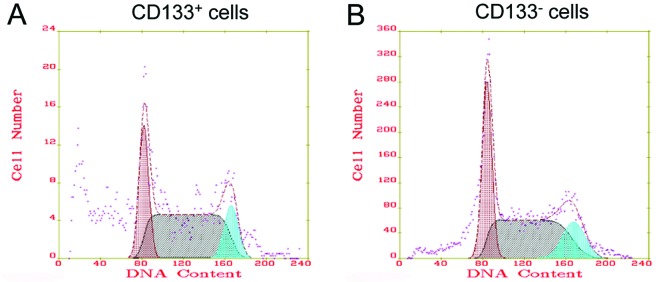
Cell cycle distribution of CD133^+^ and CD133^−^ cells. (A) CD133^+^ cells with a Pla of 61.03±11.76; (B) CD133^−^ cells with a Pla of 62.94±3.06. Brown, G1 phase; grey, S phase; blue, G2 phase.

We examined the cell cycle distribution and cell proliferation index for the CD133^+^ and CD133^−^ cells. There were 38.97±11.76% cells in the G0/G1 phase and 42.2±17.46% cells in the S phase for the CD133^+^ cells (Fig. 4A). There was no significant difference in the cell cycle distribution between the CD133^+^ and CD133^−^ cells (Fig. 4B).

### Tumourigenic potential in mice

**Figure 5:**
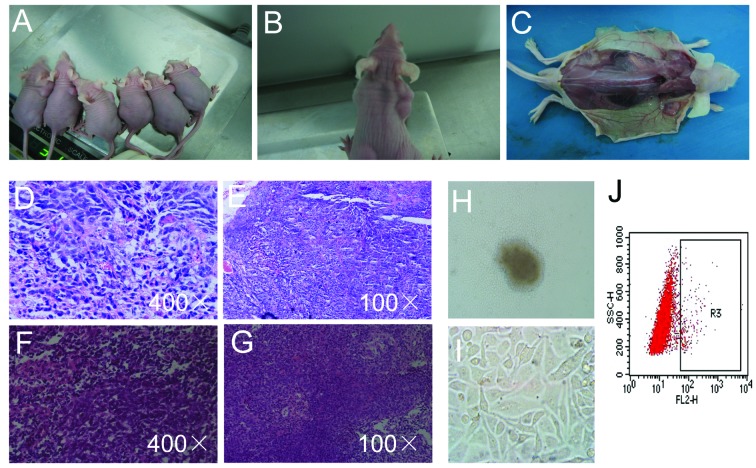
Tumour formation activity of CD133^+^ cells in nude mice. (A-C) Subcutaneous injection of CD133^+^ cells in the right dorsum of nude mice led to tumour formation; subcutaneous injection of CD133^−^ cells in the left dorsum of nude mice did not lead to tumour formation. The tissue morphology of the xenograft tumours using H&E staining: (D and E) xenograft tumours; (F and G) human NPC. (H and I) The morphology of primary xenograft tumour cells. Original magnification: ×400 in D, F and I; and ×100 in E, G and H. (J) Three independent experiments concerning the CD133 expression in primary xenograft tumour cells as detected by flow cytometry (average, 2.17±0.46%).

No tumour formation was observed in mice that received a xenograft of 1×10^4^ cells regardless of CD133 expression status. Tumour masses were found in 5 of 6 nude mice at 6–9 days after transplantation with 1×10^5^ CD133^+^ cells. The tumours grew to >1 cm in diameter in 4 of 6 nude mice at 30 days, and necrosis was found in the centre of the tumour masses. However, no tumour mass was found after the transplantation of 1×10^5^ CD133^−^ cells. The difference was statistically significant (P<0.05) (Fig. 5A-C).

We further identified that the pathologic morphology of the xenograft tumours in the nude mice was consistent with that of undifferentiated and non-keratinised human nasopharyngeal tissue. Similar to human NPC tissues, the xenografts exhibited a specific morphology with large nuclei and dark staining. They exhibited similar nuclear atypia and mitotic frequency. We found low differentiation, abundant blood vessels and some necrosis in the centres of the tumours (Fig. 5D-G). In the 5 primary cell cultures, 3 samples successfully survived and proliferated to passage. The tumour cells slowly grew out from the centre of the tissues with large nuclei and little cytoplasm at day 3. The cells grew as irregular polygons to fused large cells similar to that of CNE2 cells from an initial spindle shape. After passaging, the xenograft tumour cells took ~4 days to reach confluence in a 25-cm^2^ flask, a period that was approximately twice as long as that for the CNE2 cell line (Fig. 5H and I). There were 2.17±0.46% CD133^+^ cells in the xenograft tumour cells (Fig. 5J).

### Characterisation of the primary human NPC cells

**Figure 6:**
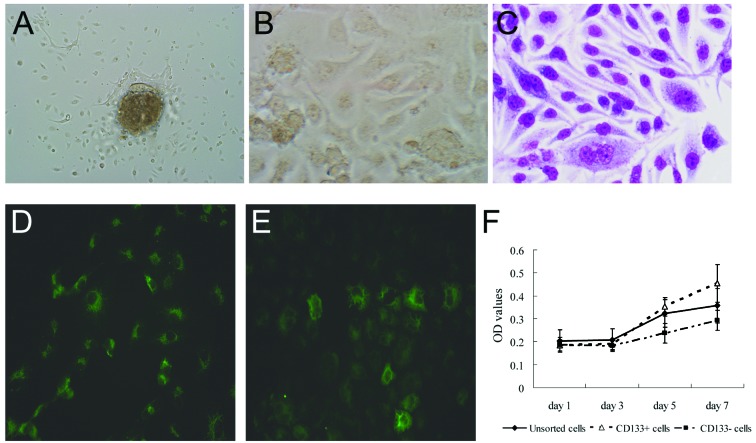
Primary human NPC cells cultured in keratinocyte-SFM medium. (A) Magnification, ×100; (B) magnification, ×400; (C) magnification, ×400 (Giemsa staining). (D and E) Cytokeratin expression in primary human NPC cells. (F) The growth curve of CD133^+^ and CD133^−^ cells in primary NPC cells.

Nine of 20 primary human NPC cell samples successfully survived and proliferated to passage. Under culture in keratinocyte-SFM medium, the tumour cells grew out from the centre of the primary tissue and adhered to the dish exhibiting an irregular polygon shape on day 3. Four samples had no passage, and 4 samples had 2–3 cell propagations. One clone with 6 propagations was used for subsequent experiments (Fig. 6A-C). The cells demonstrated expression of epithelial markers of CK (Fig. 6D-F). The expression rate of membrane CD133 was 2.8±0.17% for 3 independent experiments. Additionally, we found that the CD133^+^ cells displayed a higher proliferative capacity when compared with that of the CD133^−^ cells on days 5 and 7 (P<0.05) and compared with that of the unsorted cells on day 7 (P<0.05). Furthermore, the unsorted cells displayed a higher proliferative capacity when compared with the CD133^−^ cells on days 5 and 7 (P<0.05) (Table I and Fig. 6F).

## Discussion

The cancer stem cell theory has provided a novel and reasonable explanation for the mechanism of the occurrence and recurrence of nasopharyngeal carcinoma. In recent years, the presence of nasopharyngeal cancer stem cells has been reported in the literature. Approximately 0.3% of the tumour cells in nude mice with human NPC xenograft tumours were label-retaining stem cells (22). Wang *et al*(21) reported side population cells (SP cells) in NPC cell lines with high tumourigenic ability and some chemotherapy tolerance. However, ideal surface markers for stem cells in nasopharyngeal carcinoma remain unidentified. Our study identified that CNE2 cells contain a small population of CD133^+^ cells with a strong potential for self-renewal, proliferation and differentiation, suggesting that CD133 represents a marker of NPC stem cells.

In the present study, CNE2 cells stably expressed the CD133 antigen with an expression rate of 3.36%. In particular, CD133^+^ cells, but not CD133^−^ cells, expressed high levels of both Nanog and Sox2, marker genes of normal human stem cells. Previous research has shown that various cancer stem cells share consistent differentiation markers with normal stem cells. The CD34^+^CD38^−^ surface antigen phenotype is not only specific to tumour stem cells in acute myeloid leukaemia but also to human hematopoietic stem cells (2). CD133 and nestin have been demonstrated to exhibit identical expression levels in both glioma cancer stem cells and normal neural stem cells (23). Our results suggest that CD133 may be an ideal candidate surface marker for cancer stem cells in nasopharyngeal carcinoma. CD133^+^ cells presented a significantly greater proliferative capacity than CD133^−^ cells. More importantly, injection of 1×10^5^ CD133^+^ cells, but not CD133^−^ cells, resulted in a tumourigenicity rate of 83.3%. Pathological staining of the xenograft tumours and primary tumour cultures demonstrated that the tissues of the xenograft tumours were morphologically similar to the tissues of the pathological tumours of human nasopharyngeal carcinoma. Although culturing primary human NPC tissues was very difficult, we successfully cultured one clone of NPC stem cells in 6 NPC clones. Similarly to the results from the CNE2 cell line, the CD133^+^ cells from primary human tissues displayed a higher proliferative capacity compared with CD133^−^ and unsorted cells. Our findings suggest that CD133^+^ cells present a higher proliferative capacity. When assessing the differentiation capacity of purified CD133^+^ NPC cells, we found that purified CD133^+^ NPC cells rapidly lost the expression of CD133 after differentiation. This finding indicates that most of the CD133^+^ NPC cells differentiate to tumour cells except for a few that maintain the activity of cancer stem cells.

It has been reported that most *in vivo* cancer stem cells are in a quiescent state (G0 phase) (24). However, we found no difference in the distribution of the G0/G1, S and G2/M phases between the CD133^+^ and CD133^−^ cells, perhaps because of changes in the micro-environment (25). From the moment of separation, significant changes were noted in the micro-environment (niche) of the cells that caused the cancer stem cells to rapidly enter the cell cycle to restore the normal proportion of non-cancer stem cells (26). A previous report concerning the HEP-2 tumour cell line reported that CD133^+^ cells exhibiting a high capacity for self-renewal and strong *in vivo* tumourigenicity have a cell cycle distribution similar to that of unsorted cells (27). These data suggest that some types of cancer stem cells may be in a quiescent state.

In summary, using cell lines and primary cancer cells, we found that CD133 may be a useful surface marker for cancer stem cells of nasopharyngeal carcinoma. CD133^+^ cells possess a strong potential for self-renewal, proliferation and differentiation and exhibit high tumourigenicity.

## Figures and Tables

**Table I:** Ultraviolet absorption of CD133^+^, CD133^−^ and unsorted tumour cells in primary NPC cells.

	CD133^+^	CD133^−^	Unsorted	F-value	P-value
Day 1	0.184±0.025	0.187±0.032	0.202±0.048	1.388	0.259
Day 3	0.190±0.023	0.183±0.022	0.207±0.049	2.342	0.106
Day 5	0.352±0.041	0.237±0.043	0.323±0.059	27.650	0.000
Day 17	0.454±0.083	0.293±0.044	0.357±0.075	24.606	0.000

NPC, nasopharyngeal carcinoma.
